# Characterization of Clinical and Carrier *Streptococcus agalactiae* and Prophage Contribution to the Strain Variability

**DOI:** 10.3390/v12111323

**Published:** 2020-11-18

**Authors:** Aneta Lichvariková, Katarina Soltys, Tomas Szemes, Livia Slobodnikova, Gabriela Bukovska, Jan Turna, Hana Drahovska

**Affiliations:** 1 Department of Molecular Biology, Faculty of Natural Sciences, Comenius University in Bratislava, Ilkovicova 6, 841 15 Bratislava, Slovakia; lichvarikova.aneta@gmail.com (A.L.); katarina.soltys@uniba.sk (K.S.); tomas.szemes@uniba.sk (T.S.); jan.turna@uniba.dk (J.T.); 2 Comenius University Science Park, Ilkovicova 8, 841 04 Bratislava, Slovakia; 3 Department of Microbiology and Virology, Faculty of Natural Sciences, Comenius University in Bratislava, Ilkovicova 6, 841 15 Bratislava, Slovakia; 4 Institute of Microbiology, Medical Faculty, Comenius University in Bratislava, 813 72 Bratislava, Slovakia; livia.slobodnikova@fmed.uniba.sk; 5 Institute of Molecular Biology, Slovak Academy of Sciences, 840 05 Bratislava, Slovakia; gabriela.bukovska@savba.sk

**Keywords:** *Streptococcus agalactiae*, MLST, prophage, WGS

## Abstract

*Streptococcus agalactiae* (group B Streptococcus, GBS) represents a leading cause of invasive bacterial infections in newborns and is also responsible for diseases in older and immunocompromised adults. Prophages represent an important factor contributing to the genome plasticity and evolution of new strains. In the present study, prophage content was analyzed in human GBS isolates. Thirty-seven prophages were identified in genomes of 20 representative sequenced strains. On the basis of the sequence comparison, we divided the prophages into eight groups named A–H. This division also corresponded to the clustering of phage integrase, even though several different integration sites were observed in some relative prophages. Next, PCR method was used for detection of the prophages in 123 GBS strains from adult hospitalized patients and from pregnancy screening. At least one prophage was present in 105 isolates (85%). The highest prevalence was observed for prophage group A (71%) and satellite prophage group B (62%). Other groups were detected infrequently (1–6%). Prophage distribution did not differ between clinical and screening strains, but it was unevenly distributed in MLST (multi locus sequence typing) sequence types. High content of full-length and satellite prophages detected in present study implies that prophages could be beneficial for the host bacterium and could contribute to evolution of more adapted strains.

## 1. Introduction

*Streptococcus agalactiae*, also known as group B Streptococcus (GBS), inhabits the gastrointestinal and urogenital tracts of 35% of the healthy population. These Gram-positive β-hemolytic bacteria represent a leading cause of invasive bacterial infections in newborns, such as pneumonia, sepsis, and meningitis [1]. Neonatal GBS infections can be divided into the early-onset disease (EOD) occurring within the first week of life due to pathogen transmission from asymptomatic mother and the late-onset disease (LOD) manifested up to three months of age [2]. GBS is also responsible for invasive and non-invasive diseases in older and immunocompromised adults resulting in skin or soft tissue infections, bacteraemia, urinary tract infections, and pneumonia [3].

Typing of GBS, isolated from different countries, by multi locus sequence typing (MLST) and capsular serotyping have shown that most human carriage and clinical isolates cluster into a small number of clones and their prevalence varies over time and by geographic locations [4]. Strains belonging to MLST clonal complex (CC) 17 are responsible for the vast majority of the meningitis among neonates [5] and CC-1 has higher incidence in adult infections [6,7].

Bacteriophages including prophages are important factors in the evolution of the host genomes. Prophages are widespread and linked to increased infectivity, pathogenicity, and virulence in many bacterial species [8]. Prophage-encoded virulence factors have been identified in *Streptococcus pneumoniae* and *Streptococcus pyogenes* [9,10,11,12]. In *S. agalactiae*, lysogeny was first described in 1969, when temperate phages were isolated from strains of bovine origin [13]. Analysis of sequenced human *S. agalactiae* showed that prophages contribute to genomic diversity and phage-associated genes, accounting up to 10% of all strain-specific genes [14]. Several studies have confirmed that differences in prophage content are present between GBS serotypes and CCs [15,16]. By genome sequencing of 14 representative GBS strains, 22 prophages were detected, and these phages were distributed into 6 groups, the most prevalent group was present in 10 strains [17]. Further PCR-based detection revealed the presence of at least one prophage in 72.4% of the 275 isolates and a greater prophage content in GBS strains isolated from infected neonates and adults in comparison with colonizing strains. Prophages have also been shown to protect bacteria from horizontal gene transfer including superinfection by other phages [17]. Another study investigated properties of prophages by their induction with mitomycin C and observed that the phages from CC-17 lysogenic strains displayed lytic replication in bacterial hosts from several phylogenetic lineages, whereas phages obtained from non-CC-17 strains lysed only bacteria of similar evolutionary origin [18].

In the present study, we characterized a set of *S. agalactiae* isolates from pregnancy screening (PS) and from non-pregnant adults suffering from GBS infections. Whole-genome sequences (WGS) of representative isolates were used for identification of prophages in the genomes. Using the PCR approach, we further determined the content of prophages in a set of the 123 GBS isolates and observed correlation between sequence type and the presence of prophages. Studies on the temperate phages are important not only for determination of their virulence impact, but also as the source of phages as potential antimicrobial agents for management of GBS vaginal infections, since no lytic phage infecting GBS has been isolated to date [18,19,20].

## 2. Materials and Methods

### 2.1. Bacterial Strains and Growth Condition

*S. agalactiae* strains were isolated from clinical samples of patients during the years 2016–2018 at University Hospital (Bratislava, Slovakia) and from women during pregnancy screening between the 33th and 37th week of pregnancy by the Laboratory of Clinical Microbiology, Medirex (Nitra, Slovakia), during the year 2018. All strains were cultivated aerobically in Todd Hewitt broth (THB) overnight and Todd Hewitt agar (Biolife, Monza MI, Italy) for 48 h at 37 °C.

### 2.2. DNA Isolation and PCR

Total DNA was extracted using a DNeasy Blood and Tissue Kit (Qiagen, Hilden, Germany) following the manufacturer’s protocol for G + bacteria. Cell lysates were prepared according to [21] and used for PCR, with primers listed in Table S1. All strains were confirmed as *S. agalactiae* by PCR targeting *dltS* gene [22]. PCR-serotyping was performed according to [22], and the type of surface alpha-like protein was determined according to [23].

### 2.3. Multilocus Sequence Typing

MLST was performed by PCR and Sanger sequencing using *S. agalactiae* MLST scheme [24]. The allele numbers and sequence types (STs) were determined on the basis of *S. agalactiae* MLST database (http://pubmlst.org/sagalactiae). The goeBURST algorithm [25] was used to establish relationships between STs. Clonal complexes (CCs) were defined as single-locus variants. Minimum spanning tree showing the relationship between STs lineages was created with BIONUMERICS (Applied Maths, Sint-Martens-Latem, Belgium).

### 2.4. Whole Genome Sequencing, Sequence Analysis, and Prophage Identification

Libraries for massively parallel sequencing were prepared by using Nextera XT DNA Library Preparation Kit (Illumina, San Diego, CA, USA). Paired-end sequencing with 2 × 150 bp reads was carried out on NextSeq systems or with 2 × 300 bp on MiSeq (Illumina). De novo assembly was performed on CLC Genomics (QIAGEN Hilden, Germany; https://digitalinsights.qiagen.com) and SPAdes (Center for Algorithmic Biotechnology, St. Petersburg, Russia [26]. The obtained contigs were filtered to sequences longer than 500 bp with coverage higher than 20. Assemblies were annotated with RAST (http://rast.nmpdr.org/) [27]. A phylogenetic tree, based on 100 unique genes, was constructed in Patric (https://www.patricbrc.org/) [28]. CRISPRs finder was used to identify CRISPRs and *cas* genes (https://crisprcas.i2bc.paris-saclay.fr/CrisprCasFinder/Index) [29]. Spacer sequence identity with prophages was analyzed by BLAST. Prophage regions were detected by PHASTER web server (http://phaster.ca/) [30] combined with RAST subcategories and confirmed by manual curation of the results. RAST annotation of all prophage genes was manually corrected by HHPred (https://toolkit.tuebingen.mpg.de/#/tools/hhpred) [31] and according to search in the BLAST non-redundant database using BLASTP [32]. The predicted gene functions were assigned if E-values were bellow 10e-6 and sequence identities were at least 70%. All putative prophage sequences were analyzed manually using Geneious version 11.1.5 (Biomatters Ltd., Auckland, New Zealand), and the total number of open reading frames (ORFs), overall sequence length, and GC content were calculated in Geneious as well. A phylogenetic tree based on genome comparison at the nucleotide level was performed by the Genome-BLAST Distance Phylogeny (GBDP) method using the formula D0 at Victor web software (https://ggdc.dsmz.de/victor.php#) [33]. A phylogenetic tree of phage integrases compared at the amino acid level was performed by MEGA6 using the JTT model [34]. The number of holin transmembrane domains was predicted using TMHMM Server v. 2.0 (http://www.cbs.dtu.dk/services/TMHMM/) [35]. The whole-genome sequences of strains analyzed in this study were deposited in the *S. agalactiae* MLST database (http://pubmlst.org/sagalactiae) [36] under ID: 5586 (KMB-533), 5587 (KMB-534), 5590 (KMB-548), 5603 (KMB-562), 5605 (KMB-572), 5624 (KMB-639), 5627 (KMB-642), 5637 (KMB-659), 14111 (KMB-564), 5651 (KMB-675), 5655 (KMB-679), 5658 (KMB-682), 5974 (KMB-797), 13457 (KMB-833), 13458 (KMB-861), 5975 (KMB-884), 5976 (KMB-885), 5977 (KMB-887), 5978 (KMB-889), and 5979 (KMB-890).

### 2.5. Detection of Prophages within S. agalactiae Strain Collection

Specific PCR primer pairs (two for each prophage group) were designed (Table S1). Phage integration sites were confirmed by PCR with primers complementary to phage and bacterial attachment sites.

### 2.6. Analysis of Prophage Induction by WGS

GBS strains were cultivated 18 h in 100 mL THB broth at 37 °C; then, the cultures were centrifuged (8000× *g*, 30 min, and 4 °C). Supernatants filtered through 0.2 μm pores were precipitated by 10% polyethylene glycol and 1 M NaCl (final concentration) and the sediments were dissolved in 0.5 mL suspension buffer (100 mM NaCl, 8 mM MgSO_4_, 0.002% gelatine, 50 mM Tris-HCl). Free DNA was removed with 20 U (final concentration) of DNase I (Thermo Scientific, Waltham, MA, USA) for 1 hour and phage DNA was extracted using a Phage DNA isolation kit (Norgen, Biotec Corp., Thorold, ON, Canada). DNA was sequenced by the same protocol as was used for bacterial genome sequencing. Contigs with coverage at least 10 times higher than average coverage were further analyzed as putative phages.

## 3. Results

### 3.1. Analysis of GBS Strains

We have characterized GBS strains isolated from adult hospitalized patients and from women during pregnancy screening. A total of 73 *S. agalactiae* strains were isolated from urogenital samples of patients aged 18–89 years who were suffering from urogenital tract infections with underlying conditions of diabetes, malignancy, or liver disease. Other GBS strains were isolated during PS in a local clinical laboratory. Overall, 3802 PS samples were tested during the year 2018, and 475 (15.7%) of them were positive. We randomly selected 50 isolates (14 strains from vagina and 36 strains from recto-vaginal swab) for detailed analysis.

The clonality of strains was determined by MLST, which revealed the presence of 26 sequence types (STs) among all 123 isolates. The STs were clustered into five major clonal complexes: CC-1 (42.3%), CC-12 (9.8%), CC-17 (13%), CC-19 (8.1%), and CC-23 (13%), and eight singletons (Figure S1; Table S2).

All isolates were PCR serotyped and a type of the alpha-like family protein was determined. The results shown that the serotypes V (42.3%) and III (22.8%) were the most prevalent, followed by Ia (14.6%), II (11.4%), Ib (4.1%), IV (3.3%), VI, and VII (both 0.8%). The Alp 2/3 (46.3%), Rib (23.6%), Alpha C (20.3%), and Epsilon (9.8%) surface proteins were present in the collection. We observed a correlation between sequence types, serotypes, and surface proteins.

GBS strains isolated from hospitalized patients belonged mainly into CC-1 (55%), followed by CC-23 (15%), CC-19 (11%), CC-12, and CC-17 (5% for both). Isolates obtained from pregnant women showed higher genetic heterogeneity compared to clinical strains. The CC-1 (24%), CC-17 (24%), and CC-12 (22%) were predominant clonal complexes in these strains (Figure S1; Table S2).

### 3.2. Whole Genome Sequencing

High coverage draft genome sequences were received for 20 isolates selected to cover all main serotypes and CC (Table S3). The genome sizes ranged between 1.99 and 2.19 Mbp and annotation by RAST showed that of the genomes contained from 1989 to 2189 ORFs, approximately 85% of them had assigned function.

**Figure 1:**
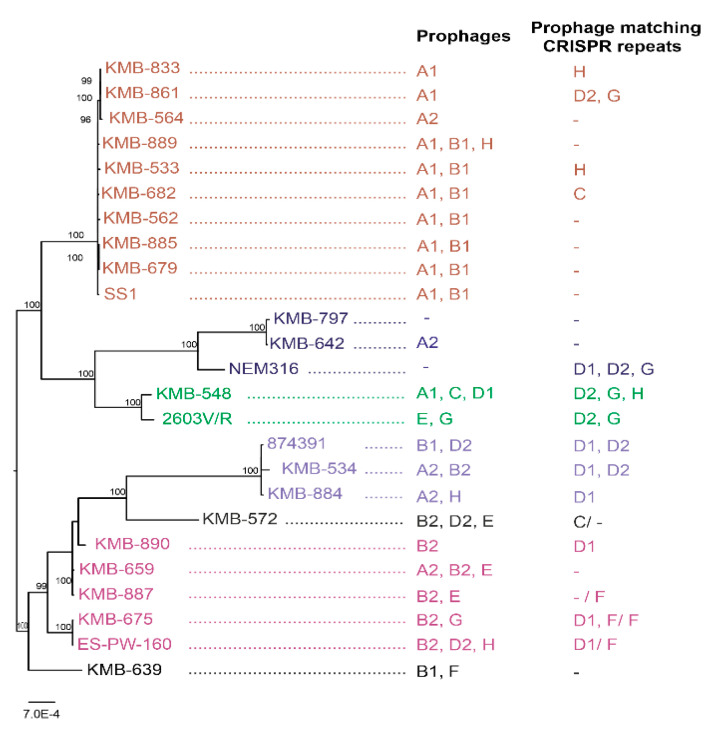
Distribution of prophages belonging to the groups A-H and CRISPR repeats complementary to these prophages in 20 sequenced group B Streptococcus (GBS) strains. Dendrogram based on comparison of 100 loci was used for genome similarity estimation with one reference genome per clonal complex (CC): SS1 (CP010867.1), NEM316 (AL732656.1), 2603V/R (NC_004116.1), 874,391 (CP022537.1), ES-PW-160 (LCVT00000000.1). Strains clustered according to CCs: CC-1 (brown), CC-12 (red), CC-17 (violet), CC-19 (green), CC-23 (blue), and sequence type (ST) not involved in CC (black).

The sequenced strains belonged to five major clonal complexes and two singleton STs; both clinical and PS screening isolates were evenly represented. On the basis of the genome comparison, we separated the strains into clusters corresponding to their clonal complexes. Multiple strains belonging to CC-1 and the strain pairs belonging to CC-17 and CC-23 showed a high degree of similarity, whereas four strains from CC-12 were much more heterogeneous (Figure 1; Table S3).

We analyzed presence of virulence genes in genomes and observed clustering mainly according to clonal complexes. The detection of five adhesins showed frequencies of *fbs*A, *fbs*B, *lmb*, *bib*A, and *ssr*1 to be 100, 70, 95, 60, and 55%, respectively. All strains contained one or two pilus genomic islands; the most frequent combination was PI-1/PI-2a—the PI-2b island was present only in CC-17. All strains possessed *cyl* cytolysin operon, the *cfb* encoding for CAMP factor, *sod*A, and *pon*A virulence genes; the *scp*B and *hyl*B were missing in three or two strains, respectively. The detection of antibiotic resistance genes showed a high prevalence of tetracycline resistance (*tet*(M), *tet*(O), *msr*D, *mef*A, 85%) and macrolide resistance genes (*erm*(A), *erm*(B), *erm*(T), 70%); two strains possessed genes encoding for aminoglycoside resistance (*ant*(6)-Ia, *sat*4A, *aph*(3’)-III, *cat*) (Table S3).

### 3.3. Prophage Detection in S. agalactiae

**Figure 2:**
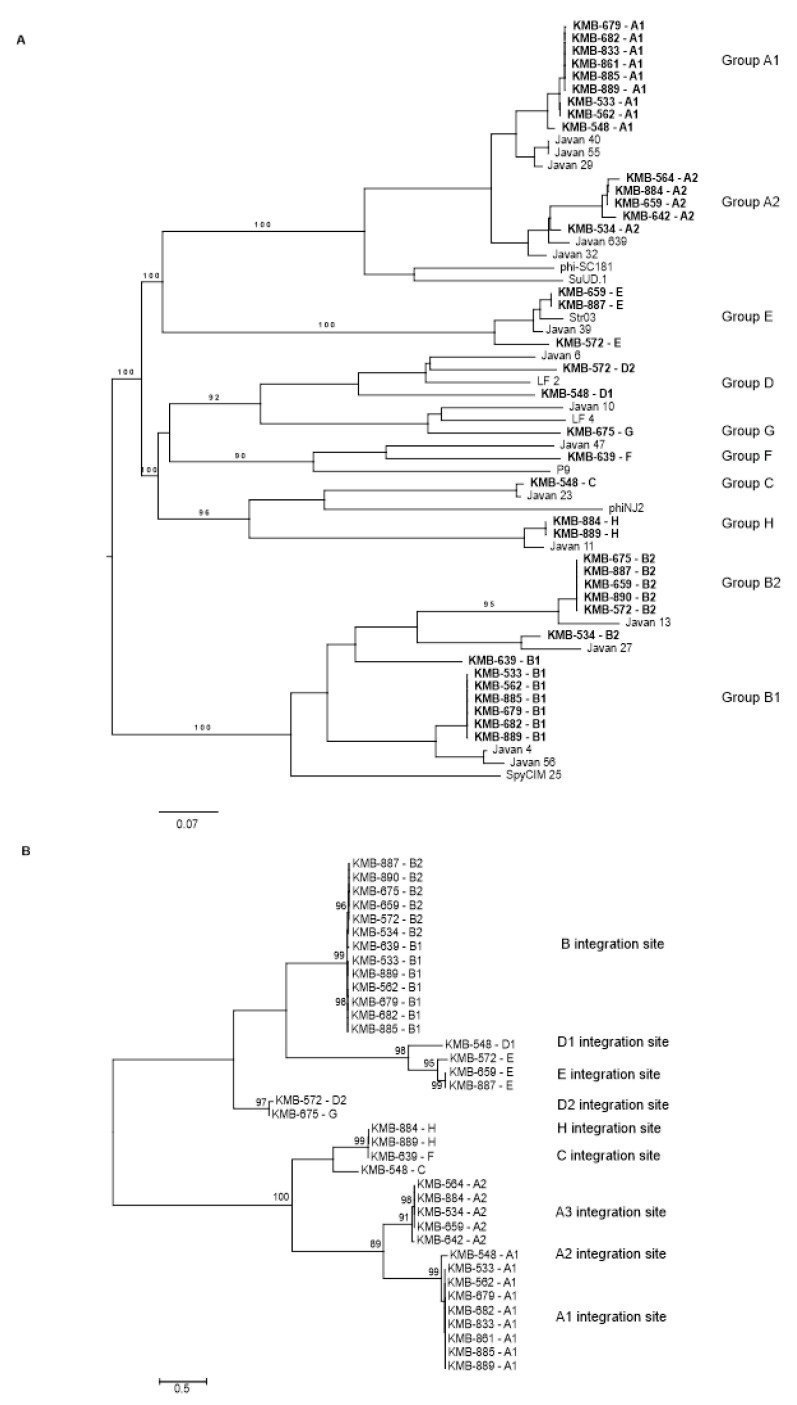
Phylogenetic tree of GBS prophages identified in the present study: (**A**) genome comparison at nucleotide level performed by the Genome BLAST Distance Phylogeny (GBDP) method using the formula D0 available at Victor web software [33]; (**B**) maximum likelihood tree comparing phage integrases at amino acid level in MEGAX using Jones–Taylor–Thornton (JTT) model [34].

**Figure 3:**
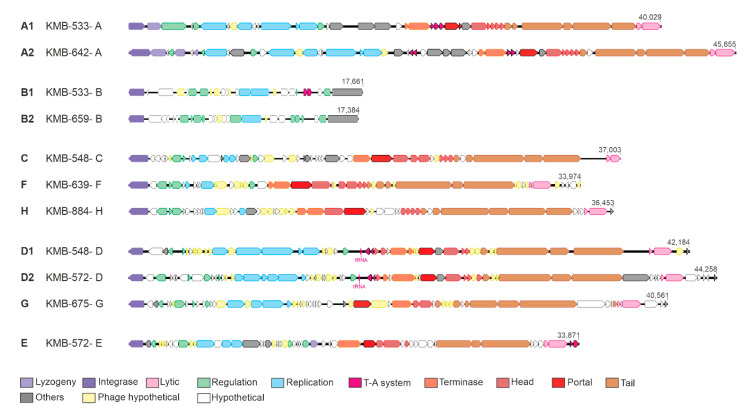
Genome maps of representative prophages belonging to A–H groups.

By combination of several in silico approaches, we detected 37 prophages in 19 sequenced strains; one CC-23 isolate did not contain any phage. One to three prophages per bacterial genome with the size 16–45 kbp were present. On the basis of the sequence comparison, we divided the prophages into eight groups named A–H (Figure 2A). This comparison corresponded to relationships based on phage integrases (Figure 2B). All prophages, except group B, represented full-length prophages possessing structural genes enabling morphogenesis of a phage virion (Figure 3; Table 1; Table S4).

Prophages from group A were detected in 14 strains. These phages were of 38–45 kbp long, encoding 41–53 genes. Group A phages showed more than 90% mutual similarity along 64–100% genome coverage. Two subgroups could be distinguished according to the length of the homologous regions and integrase similarity (Figure 2). The cluster A1 contained prophages from CC-1 and CC-19 strains; A2 prophages were present in CC-12, CC-17, and CC-23, and in one CC-1 strain. Group A prophages were integrated to at least three different sites: between N-acetyl-diaminopimelate deacetylase and PrkC protein kinase, upstream from Rib protein gene, and upstream from a HAD family hydrolase gene (Figure 2; Table S4).

Group B prophages of 16–18 kbp containing 24–29 genes were detected in 13 strains. These elements represented satellite prophages as they lacked structural genes for head and tail morphogenesis (Figure 3). In group B prophages, high DNA similarity was observed in more than half of genome length. The other part separated B prophages into two subgroups which correlated with clustering of the host strains; B1 group was present in CC-1 and ST-6, and B2 prophages were integrated in CC-12, CC-17, and ST-130 strains. All B prophages possessed the same integration site in S4p ribosomal protein.

Except for the frequently identified groups A and B, 10 rarely occurring prophages were also detected. According to their DNA similarity, they could be divided into three clusters. The first cluster contained four prophages from groups C, F, and H with the size of 33–36 kbp. These prophages showed high similarity in integrase genes, but two different integration sites. Three phages were inserted into the *com*GC gene and one phage was integrated into transcription regulator of copper transport operon (Table S4).

Related D and G groups contained three prophages with the size of 38–44 kbp. Two distinct attachment sites were revealed for these prophages, D1 site was in tRNA ^ser^ gene whereas D2 and G sites were flanked by competence-specific sigma factor *com*X and the histidine phosphatase gene.

Three prophages assigned into E group were 31–34 kbp long and were integrated into tRNA ^cys^ gene (Figure 2; Table 1).

All prophages detected in our study were significantly similar (>94% identity covering at least 63% of the entire prophage sequence) to in silico-identified prophages from GBS genomes deposited in DNA databases (Figure 2A) [10] and to several *Streptococcus* phages; group A prophages were relative to phi-SC181, phi-SsUD.1, phi-m46.1, and phiD12 phages [37,38,39,40]; the B satellite prophages were related to the phage-like chromosomal islands from *S. agalactiae* and *S. pyogenes* [41]. Low-level of similarity was detected for C and H prophages with *S. suis* phiNJ2 and prophage F with *S. equi* P9 phage [40,42]. Bacteriophages from groups D and G were relative to *S. agalactiae* phages LF2 and LF4 [20]. Group E bacteriophages were similar to *S. agalactiae* phage LF3 [20] and *S. pyogenes* phage Str03 [43] (Figure 2A; Table S4).

### 3.4. CRISPR-Cas Detection in S. agalactiae Genomes

Presence of CRISPR-Cas systems was analyzed in sequenced strains. The 2-A CRISPR1 type was detected in 17 strains, and along with this system the 1-C CRISPR2 was further found in 4 strains. Three strains lacked any CRISPR-Cas. The number of spacers ranged from 1 to 19 for 2-A CRISPR1 and from 4 to 12 in 1-C CRISPR2 (Table S5).

Overall, 157 different spacers were detected in all genomes, with 24 spacers repeatedly present in several strains. CC-1 strains in particular shared conserved terminal spacers, but the leader ends were unique for each strain. Among CC-12, the 2-A CRISPR1 repeats were heterogeneous with unique spacers, but 1-C CRISPR2 shared similar spacer pattern at trailer end (Table S5). We did not observe a correlation between number of CRISPR spacers and prophage numbers in strains.

Nineteen spacers matched perfectly or imperfectly (>93% sequence identity) to prophages identified in the present study. Surprisingly, no spacer showed similarity to A, B, and E prophages. The groups C, F, and H were targeted in six strains, and spacers complementary to the groups D and G were detected in seven strains (Figure 1, Table S5).

### 3.5. PCR-Based Prophage Identification

The lysogeny status of all 123 strains from our collection was tested by PCR. Two conservative genes were selected for each prophage group as detection markers, and prophage integration in genome was detected by primers overlapping *att*B site for some prophages. Presence of at least one prophage was observed in 105 isolates (85%). The prevalence of phage carriage among GBS strains varied according to prophage group from 1.6% for group F to 71% for group A. Prophages were detected in clinical as well as PS isolates (Table 1; Table S2).

**Figure 4:**
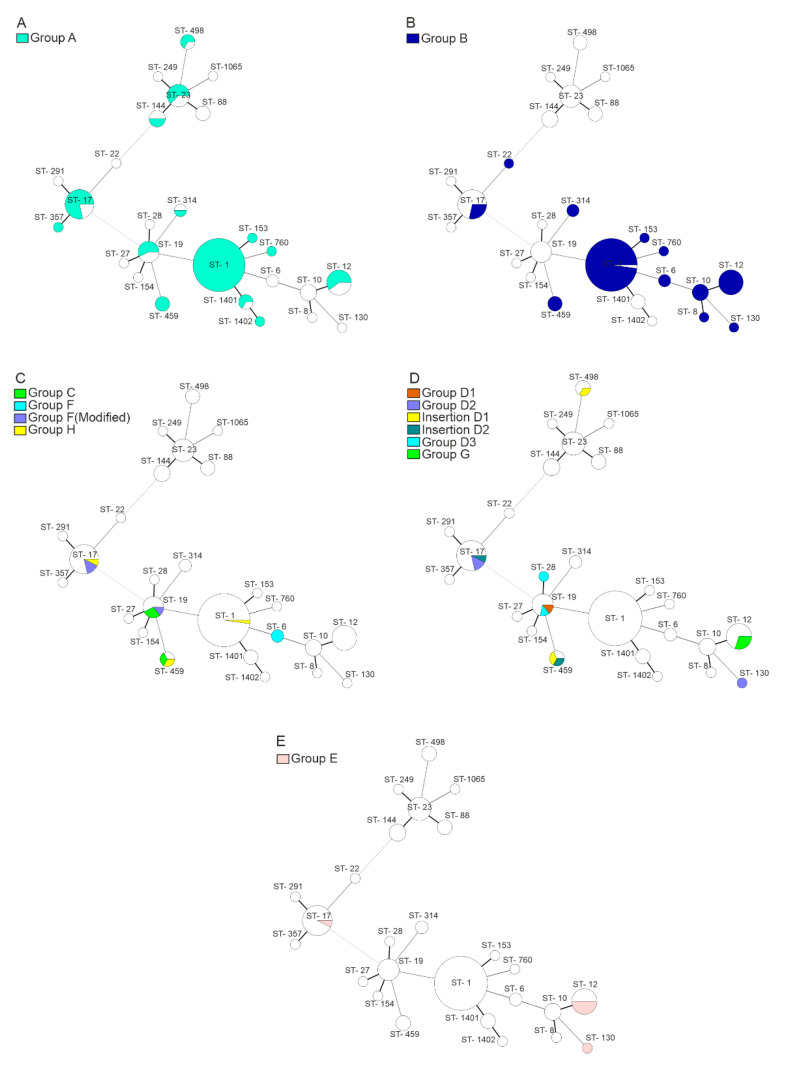
Distribution of prophage groups according to multi locus sequence typing (MLST) sequence types in 123 GBS strains: (**A**) group A; (**B**) group B; (**C**) related groups C, F and H; (**D**) related groups D and G; (**E**) group E.

The high prevalence of prophage groups A and B was observed. The prophages A were present in 87 from 123 strains (71%). This prophage was associated with isolates belonging to CC-1 (51 from 52 isolates), and it was also present in approximately half of strains belonging to other CCs and singleton ST (Figure 4). Prophages from B group were detected in all CC-12, frequently in CC-1, and to a lesser extent in CC-17 strains, while CC-19 and CC-23 lacked this element. Prophages from other groups were detected infrequently. Markers of the C, F, and H prophages were detected in seven isolates. Three other strains possessed *fib*F marker but lacked *ptl*F. Five strains were positive for D1, D2, and G prophages, but another six strains were positive for PCR tests partially—four strains had a D1 or D2 integration site occupied by some unknown element and two another isolates possessed *fib*D and *ptl*D markers integrated in an unknown genome site (named D3). Prophages of group E were present in seven strains, with the majority of them belonging to ST-12 (Figure 4).

### 3.6. Prophage Induction

Functionality of prophages was tested by the phage presence in culture medium supernatants. Whole DNA sequencing of isolated phage particles revealed six contigs with coverage at least 10 times higher compared to average bacterial DNA that corresponded to six released phages from five strains. We confirmed induction of group A2 prophages from *S. agalactiae* KMB-642 and KMB-659, group C prophage from *S. agalactiae* KMB-548, group F prophage from *S. agalactiae* KMB-639, and two prophages (D2 and E) from *S. agalactiae* KMB-572 (Table S6).

## 4. Discussion

*Streptococcus agalactiae* is a commensal bacterium colonizing the gastrointestinal and genitourinary tract, but it is also the causative agent of serious neonatal infections and adult infections. The aim of this study was to characterize GBS strains from Slovakia and to compare isolates from adult infections with those obtained from pregnancy screening. The study was focused on prophage variability because prophages can significantly contribute to the microevolution of bacterial hosts.

We analyzed PS strains from Slovakia obtained during 2018. The observed prevalence of GBS 15.7% was lower than that obtained in other European countries [44]. The serotype and ST distribution in 50 randomly selected strains was similar to the frequency observed in other countries. We found a slightly higher incidence of serotype V (covering mainly CC-1 strains) and II (CC-12) compared to other reports [45,46]. Serotype III (30%) and the serotype III-associated CC-17 (24%), which are responsible for a significant proportion of GBS neonatal diseases, were slightly higher than the world average, but comparable to other European populations [44,47].

GBS collection was supplemented by 73 strains from hospitalized adult patients. Strains were isolated from non-invasive urogenital infections, most of them from urine (80%) and vaginal swab (12%). The high prevalence of serotype V and CC-1 strains (55%) and under-representation of CC-12 and CC-17 in clinical set were the main distinctions from the pregnant carrier strains. The proportion of CC-1 in our collection was higher compared to a recent study on a GBS invasive population from the USA [7] and Taiwan [6], but not as high as in the study from Houston and Toronto [48]. The reason for the variations may be due to the different types of infections being investigated or the geographical distance. The high prevalence of GBS serotype V in adult urinary tract infections was also detected by [49]. Studies [50,51] found that the ratio of serotype V increased with the patient age. However, this was not true for our collection. Another interesting difference between our study and other studies was the predominance of serotype II (87%) in both clinical and PS CC-12 strains, while serotype Ib was predominant in in Taiwan [6] and Canada [52].

Using the whole-genome sequencing of 20 representative GBS genomes, we observed great differences in prophage content, even between strains belonging to the same ST. These results were confirmed by PCR detection of prophages in all strains from our collection. The average number of prophages reached 1.5 per bacterial genome—the highest prophage content was present in CC-12 and CC-1 (2.2 and 1.9), and the lowest level 0.43 per genome was in CC-23. These values correspond with distribution of the prophage groups according to the sequence types (Figure 4) and are comparable with the study [17].

In our GBS, the group A prophages were the most frequent. Similar prophages were often found in other studies, e.g., prophages A from the study of [17], phiD12-related phages studied by [53], and several Javan phages (e.g, Javan29, Javan32, Javan40, Javan55) [10]. However, each prophage contained some unique parts in the genome, as the coverage of the most similar prophages from the database to our sequences reached only 77–86%. Group A was separated into two subgroups, A1 and A2, which differed by gene content, the integration site, the host range, and the ability of induction. Most A1 prophages were present in CC-1 and were not induced during the stationary growth phase of the host bacteria. Group A2 prophages were detected in strains belonging to four different clonal complexes. Phage induction was observed in two from the three strains tested (Table 1; Table S6). An increased prevalence of group A prophages in CC-1 strains was also observed in the study [17].

Group B prophages were also frequently detected in our collection. These satellite prophages are associated with the phage-like chromosomal islands from *S. pyogenes* and other streptococci [41]. All prophages from *S. agalactiae* group B were integrated into S4p ribosomal protein. This protein has an essential role in ribosome function, and thus its transcription is unimpeded by prophage integration. We assumed that B prophages were relatively stable in the genome because their presence and distribution of subgroups B1 and B2 almost completely correlated with ST (Figure 2). The only exception was strains from ST-17 which showed variable presence of B prophages. No induction of B prophages was observed (Table 1; Table S6), and this result also supports the assumption of the element stability.

In addition to major groups A and B, we detected some other prophages with much lower frequency. These prophages were classified into three major groups; they were fully characterized in silico and the phage induction was confirmed for four phages. Overall, 21% strains contained at least one marker of these phage elements (Table 1).

The first cluster covered prophages belonging to groups C, F, and H. With the only exception, they were inserted into *com*G operon, involved in host competence, and were similar with the phages labeled C and F in the study [17]. Insertional inhibition of transformation genes through mobile genetic elements has been described in many bacterial species and is explained as an evolutionary advantage for the element maintenance in the genome [54]. Genetic switch in *com*K gene due to the temperate phage excision has been shown to induce *Listeria monocytogenes* transformation machinery that promotes phagosomal escape and virulence [55]. This system is similar to the activation of *mut* genes by SpyCIM1 in *S. pyogenes* [41] and therefore analogous mechanisms could also be expected in GBS, but it needs to be experimentally confirmed. It is interesting that two H prophages shared high level of DNA similarity (99.98% identity covering 100% of prophage sequence) and the identical sequence of phage integrase, but the prophages were integrated into different genome locations—*com*G and *cut* genes (Figure 2B; Table S4). These prophages were present in different STs, which could be responsible for distinct integration sites. The same 21 bp conserved sequence located upstream from integrase gene in both H prophages was detected, which we propose to be the phage attachment site. By broad screening of GBS genomes [56] identified this integrase in *com*G gene only. However, the same authors also observed two different genome localizations of prophages possessing an identical transposase gene.

The prophages belonging to subgroups D2 and G were inserted into the *com*X gene encoding the master regulator of competence in streptococci. The same locus is frequently targeted by transposable elements in streptococci with possible impact on the induction of transformability [57]. Integration sites localized in *com* genes were also observed for *S. agalactiae* prophages in previous studies [10,17,56]. The tRNA genes were detected as integration sites for prophages of groups D1 and E. These sites are frequently used by bacteriophages, genomic islands, and other mobile elements [58].

Temperate bacteriophages of several pathogenic organisms affect virulence since they carry genes encoding toxins [8]. No such genes have been identified in *S. agalactiae* prophages [17,18,20]. However, some of the analyzed prophages contained genes that likely contribute to virulence and bacterial fitness (Table S4). Prophages of groups A, B, D, and E contained components of a type II toxin–antitoxin system. This system may function as a mechanism for the maintenance of temperate phage in the bacterial genome but also as defense against infection by other phages, for increased biofilm formation, persistence, and overall stress response [59,60]. Group A and D prophages contained gene-encoding Clp protease, which may affect balance between lytic and lysogenic life cycle of the phage as well as influence host virulence. Group A and C prophages possessed DNA cytosine methyltransferase, which is usually part of restriction modification systems that provide beneficial function to prophage or its host [61]. This orphan methylase protecting bacterial cells from foreign DNA invasion has been identified in various phages [17,18,40,62]. Antibiotic resistance genes are often associated with phages in various streptococcal species, including phi-SC181, phi-SsUD.1, phi-m46.1, and phiD12 phages [37,38,39,40]. However, no prophage identified in present study possessed a gene with homology to known antibiotic resistance genes.

Complementary to the detection of prophages, we studied the CRISPR-Cas systems in 20 sequenced genomes. We observed great variability in CRISPR arrays between strains even in that belonging to the same STs with no identical arrays. This corresponds with other studies [63] and allows for the use of the CRISPR sequencing as a sensitive typing method [64,65]. By comparing sequence similarity of all CRISPR spacers with A-H prophages, we observed that 11 strains (55%) possessed spacers complementary to prophages belonging to infrequently occurring groups C-H (Figure 1; Table S5). The CRISPR-Cas system could therefore be one possible reason for the low spread of these phages in GBS genomes. Surprisingly, no spacer showed similarity to frequently identified group A prophages, despite them being full prophages that are capable for induction [17,18]. The mechanism of this observation deserves further study.

Typing of GBS isolated from different countries have shown that most of the human carriage and clinical isolates cluster into a small number of clones. In this study, we analyzed a collection of GBS strains from Slovakia and we found a similar distribution of CC. High incidence of CC-1 has been found in strains isolated from hospitalized patients. However, similar strains were detected in clinical and PS collections. High content of full-length and satellite prophages was detected in GBS, which implies that prophages could be beneficial for the host bacterium. However, lytic phages capable of infecting this emerging pathogen have not been described to date. On the basis of further research, induced prophages or prophage proteins with antimicrobial activity could be used for phage therapy and decolonization of pregnant GBS vaginal carriers in the future.

## Figures and Tables

**Table 1:** Prophages in GBS sequenced genomes.

Prophage Group	Sub-Groups	No. of Prophages in Sequenced Genomes (%)	Genome Size (kbp)	GC Content (%)	No. of Genes	Phage Status	Integration Site	Induction	No. of Prophages in All Strains (%)
**A**	A1	9	39.9–40.6	42.6	40–42	complete	A1, A2	no	87 (70.7)
	A2	5	38.7–45.7	43	42–53	complete	A3	yes, no	
**B**	B1	7	16.2–17.7	35.7	24–29	satellite	B	no	76 (61.8)
	B2	6	17.4–17.9	34.5	27–29	satellite	B	no	
**CFH**	C	1	33.2	35.4	45	complete	C	yes	3 (2.4)
	F	1	34	37	49	complete	C	yes	2 (1.6) ^2^
	H	2	36.5	39.7	53	complete	C, H	NT ^1^	3 (2.4)
**DG**	D1	1	42.2	38.7	49 + 1tRNA	complete	D1	no	1 (0.8) ^3^
	D2	1	44.2	36.3	65 + 1tRNA	complete	D2	yes	3 (2.4) ^3^
	G	1	38.6	37.1	60	complete	D2	NT	3 (2.4)
**E**	E	3	32.4	40	50	complete	E	yes, no	7 (5.7)

^1^ Not tested; ^2^ presence of prophage F-specific gene fib but not complete prophage was detected in three strains; ^3^ presence of prophage D marker genes or occupation of prophage D integration site but not complete prophage was detected in six strains.

## Supplementary Materials

The following are available online at https://www.mdpi.com/1999-4915/12/11/1323/s1: Figure S1: Distribution of clinical and pregnancy screening strains and serotypes according to sequence types in 123 GBS strains. Table S1: List of primers. Table S2: GBS strains used in the study. Table S3: Sequenced GBS genomes. Table S4: Characterization of prophages from sequenced GBS genomes. Table S5: CRISPR-Cas systems in present sequenced GBS genomes. Table S6: Prophage induction.
